# Problems of problem-based learning: Towards transformative critical pedagogy in medical education

**DOI:** 10.1007/s40037-018-0489-7

**Published:** 2019-01-10

**Authors:** Alice Cavanagh, Meredith Vanstone, Stacey Ritz

**Affiliations:** 1 Health Policy PhD Program, McMaster University, Hamilton, ON Canada; 2 Michael G. DeGroote School of Medicine, McMaster University, Hamilton, ON Canada; 3 Department of Family Medicine, McMaster University, Hamilton, ON Canada; 4 McMaster Program for Education Research, Innovation & Theory (MERIT), Hamilton, ON Canada; 5 Department of Pathology and Molecular Medicine, McMaster University, Hamilton, ON Canada; 6 Medical Sciences Division, Northern Ontario School of Medicine, Sudbury, ON Canada

**Keywords:** Critical pedagogy, Problem-based learning, Undergraduate medical education

## Abstract

Problem-based medical education is based in a biomedical worldview that works to entrench deterministic ways of thinking about socioculturally-influenced health disparities in the minds of medical trainees. This perspective paper considers the utility of Paolo Freire’s critical pedagogy as a means of redressing this issue, as it may enable medical learners to perceive and address the social sources of illness that shape their patients’ lives. With an eye to advancing health equity, and educating health professionals who are responsive to marginalized and vulnerable communities, this paper considers how a problem-*posing *medical education could redefine physicians’ relationships to knowledge, identity, and to their patients.

Calls to address health inequity through medical practice have spurred a search for new approaches to professional training with the potential to shift medical practice closer to the humane, political, *social* medicine described by Virchow in the nineteenth century [1]. These efforts have achieved limited success: although ‘health advocacy’ has been recognized as a core competency for medical learners [2–4], the scope of responsibilities attached to this role remain unclear to educators and trainees [5, 6]; although commitments to social accountability are common in medical schools’ formal mandates [7], medical students are taught to be ‘aware’ of health disparities without understanding political realities that give rise to them [8]. While perspectives from critical pedagogy have not made significant inroads in medical education, we argue that Paolo Freire’s model of problem-posing education has much to offer contemporary medical educators. Acknowledging the influence of sociocultural, political, and environmental determinants on human health, Freire’s critical pedagogy could represent a pivotal intervention in efforts to engage trainees in using their emergent professional power in service of social justice.

## Introducing the ‘Pedagogy of the Oppressed’

Fifty years after its initial publication, Freire’s *Pedagogy of the Oppressed* remains a vital force in contemporary education. At the heart of Freire’s work lies a radical proposition: education must become a ‘practice of freedom’ that develops ‘critical consciousness’ amongst its learners [9]. For Freire, this means rejecting the discipline and rote memorization that characterize conventional ‘banking’ approaches to education, in which teachers didactically ‘deposit’ knowledge into the minds of their students. Instead, Freire imagines a ‘problem-posing education’ (PPE) that asks students to engage in self-directed inquiry, exploring conditions responsible for social inequities [9]. To this end, Freire imagines classrooms that upend traditional hierarchies, where students and teachers ‘teach each other, mediated by the world’ [9]. According to Freire, this egalitarian reconfiguration helps learners dispel the perception that they are isolated from their communities, or are unable to effect change; rather, students come to view themselves as capable agents of learning and action, holding a share of responsibility for the fate of their communities [9].

Although *Pedagogy of the Oppressed *has resonated with scholars over decades, critics have long argued that Freire’s focus on poverty works to obscure other types of oppression that can result in poor health outcomes [10–12]. In *Teaching to Transgress,* bell hooks suggests that educators can address this deficit by directing students to interrogate the overlapping aspects of their identity that shape their passage through the world [13–16]. We contend that this reinterpretation of Freire’s work has much to offer medical educators and learners: akin to the banking model described by Freire, medical education has long been charged with tacitly socializing students to stress patients’ personal responsibility for poor health, without acknowledging the social contexts that shape their ‘lifestyle choices’ [17, 18]. We are aware that implementing Freire’s ‘pedagogy of the oppressed’ for a social group as privileged as trainee-physicians may have its own challenges [19–21]. However, by asking medical students to think critically about social patterns of professional exclusion and the structural causes of ill health, we see fertile opportunities for fostering learners’ critically reflexive commitments to professional advocacy.

## Problem-based and problem-posing pedagogies for medical education

Freire’s problem-posing approach to education was not the only radical intervention in education to emerge during the late 1960s. First implemented at McMaster University in 1969 [22], the problem-based learning (PBL) model for medical education emerged as an answer to the accelerating pace of innovation in healthcare. Advocates for PBL argued that structuring medical learning around unique, progressing case studies prepared new physicians ‘to keep up with changing concepts and new knowledge’ [23], developing skills for self-directed research and life-long learning. Fifty years later, problem-based curricula have been implemented in training programs for healthcare providers worldwide [24–26]. Meta-analyses and systematic reviews plentifully support PBL as a model for medical education [22, 27, 28], and the competence of graduates of problem-based medical programs is comparable to their conventionally trained colleagues [29]. Problem-based pedagogies have been credited with training physicians who are better critical thinkers [30], more effective working in teams [31], and more attuned to public health concerns that arise in their clinical practice [32].

PBL and Freire’s PPE share more than a passing resemblance: both pedagogies renounce student passivity and position responsive, self-directed inquiry at the heart of their curricula; both pedagogies reject lecture-based content delivery, and re-imagine the role of teachers as supportive facilitators of learners’ exploration. In instrumental ways, however, the two approaches diverge, resulting in profoundly different implications for medical education. Below, we offer three examples comparing problem-posing and problem-based approaches to medical learning that illustrate the value of incorporating Freire’s pedagogy into medical education.

## Example one: reconceptualizing problems

One central point of divergence between PBL and PPE lies in how each model conceptualizes the ‘problems’ at the heart of each pedagogy [33]. Week-to-week in PBL, problems come in the form of short case vignettes, briefly sketching the story of a presenting patient and their medical history. These case-studies serve a dual purpose, prompting students to set medical knowledge learning objectives in order to understand each case while enlisting them into a ‘cognitive apprenticeship’ that acculturates them ‘into the thinking practices of medicine’ [34]. This framing—of individual patients as biomedical problems, largely abstracted from their family or life circumstances—by definition casts trainee-physicians in the role of problem-solvers. Although PBL destabilizes the hierarchy between learners and teachers, it does little to problematize how the power of professional authority shapes the clinical encounter or the narrow capacity of allopathic medical interventions to address structural causes of ill-health [35]. By contrast, in a PPE approach to medical learning, students would be tasked with problematizing political contexts that give rise to population health disparities, and learning about collaborative approaches to patient care [33]. Through PPE, the role of care provider could be reconfigured from that of ‘most responsible problem-solver’ to one of multiple situated actors with insight and agency, promoting collaborative engagement with patients [35].

In a case study featuring a diabetic patient who is ‘non-adherent’ to treatment, for instance, PPE tutors would help students interrogate social contexts shaping patients’ follow-through with prescribed treatment regimens, in addition to biomedical implications of poor glycaemic control [36, 37]. Asked to explore how their professional and personal identities shape clinical encounters, students would also be encouraged to forge habits of reflexive practice that last throughout their careers [38]. Practised in critiquing the origins, omissions and implications of medical epistemology from their earliest days of training, PPE-trained physicians would graduate medical school well-equipped to identify the origins of ill-health in their communities and to advocate for policy change.

## Example two: reconceptualizing knowledge

Another point of tension between PPE and PBL lies in the relationship each understands between knowledge and the knower. In PBL, students learn foundational medical knowledge through self-directed research [23], using principles of evidence-based medicine (EBM) in their evaluation of sources [39]. Although PBL has been lauded for its utility teaching critical research appraisal and responsive inquiry [39], it does not encourage students to think critically about assumptions of objectivity and generalizability that support the supremacy of scientific knowledge over other ways of approaching health problems [40]. In *Pedagogy of the Oppressed*, Freire presages this critique, writing that science becomes an instrument of oppression when its internal logics—the basis for its authority—are obscured as ‘unquestionably powerful’ [9]. In EBM, after aggregated studies are ranked according to hierarchies of value, research findings are reformulated as guidelines for broad application in the clinical context; through this process, biomedical ‘fact’ is alienated from the circumstances in which it was generated. Scientific knowledge becomes sedimented, and increasingly resistant to challenge through the compounded force of its scientific authority [40]. Later, when medical learners are asked to research cases relying only on sanctioned sources of knowledge, facets of human experience that are not accounted for in biomedical inquiry may be invisible to these learners.

Randomized controlled trials of treatment for depression that exclude gender-based analyses offer one example of this phenomenon at work: research can meet methodological criteria to be deemed ‘high quality’ according to the principles of EBM, while still obscuring gendered differences in depression symptomatology and treatment effects that have profound implications for clinical practice [41]. In PPE, therefore, students would be tasked with considering the social and political implications of how biomedical knowledge is constructed, including the legacies of medical sexism, racism and ableism entrenched in historical and contemporary medical research practices [42, 43]. Trained to consider biomedical research as socially constructed and subject to continual revision even as they use it in their practice, graduates of problem-posing medical training would be well-equipped to develop research agendas that account for knowledge as a form of power and that harness that power in service of social justice.

## Example three: reconceptualizing patients

A final point of difference between PBL and PPE lies in the use of social determinants of health frameworks to understand the lives and ill-health of patients at the heart of medicine. In PBL, the social determinants of health are often invoked to encourage students to consider how a patient’s individual characteristics might be shaping their illness [16, 44]. Although this analysis affords students an opportunity to consider the epidemiology of illness and how it aligns with positions of social inequity, case descriptions meant to inspire these conversations fall well short of matching the appreciable texture of real patients’ social identities. MacLeod’s analysis of a second year PBL medical curriculum, for instance, found that cases seldom articulated the race of patients unless the stated medical learning objectives were directly linked to racialized genetic risk factors for illnesses such as sickle cell anaemia or Tay-Sachs disease [45]. This approach leaves students with little opportunity to explore harms to health borne of racism, marginalization, and lack of access to culturally relevant healthcare services. The absence of thoughtfulness about these identity features fuels assumptions that embodied health inequities are trans-historical or biologically inevitable rather than fluid, evolving, and forged by oppressive politically imposed structures [44]. Underscoring all this lies the reality that when PBL students are tasked with deciphering a case, understanding the social context of illness often falls second to mastering biomedical learning objectives. The social context becomes something to ‘recognize’ or ‘acknowledge’ as opposed to a site of possible professional intervention or advocacy.

By contrast, students in PPE classrooms would be encouraged to probe the structural causes of ill health they see embodied in the clinic, questioning how their professional and personal roles, as well socio-political structures, are implicated in upholding these disparities in health. Students would be tasked with developing a richer understanding of how lived experiences of race, gender, migration status and other facets of social identity combine to affect their patients. Ultimately, students in a PPE classroom would challenge deterministic conceptions of health, coming to see their whole social reality as contingent—a site of activist intervention, in solidarity with the sickest in society.

## Moving towards a problem-posing medical education

While decades of research have established some of its benefits, PBL remains enmeshed in a biomedical healthcare system that maintains deterministic conceptions of health inequity and social injustice; here, we have advanced a vision of a problem-posing medical pedagogy that broadens the scope of PBL, training physicians who are actively engaged in dismantling oppressive social structures that make their patients sick. To achieve this end, students of PPE would be asked to complicate their relationships to biomedical knowledge, reconsider hierarchical dynamics in the clinic, and re-centre the struggle against social causes of illness at the heart of their future practices of medicine and medical research. Although Freire’s pedagogy remains beyond the boundaries of medical education as it is imagined today, pressing challenges to human health make the embrace of this—and other—transformational pedagogies both needed and necessary.
